# Lifetime Evaluation of Photovoltaic Polymeric Backsheets under Ultraviolet Radiation: From Chemical Properties to Mechanical Modeling

**DOI:** 10.1021/acsomega.2c06424

**Published:** 2022-12-03

**Authors:** Jia-Wei Zhang, Kai Feng, Sombel Diaham, Qiang-Qiang Liao, Xing Zhou, Chatchai Putson

**Affiliations:** † School of Electrical Engineering, Xi’an University of Technology, Xi’an, Shannxi710048, China; ‡ Université de Toulouse, UPS, INPT, LAPLACE (Laboratoire Plasma et Conversion d’Energie), 118 route de Narbonne, F-31062Toulouse cedex 9, France; § Shanghai University of Electric Power, Shanghai200090, China; ∥ Faculty of Printing, Packaging Engineering and Digital Media Technology, Xi’an University of Technology, Xi’an, Shannxi710048, China; ⊥ Materials Physics Laboratory, Department of Physics, Faculty of Science, Prince of Songkla University (PSU), Songkhla90112, Thailand

## Abstract

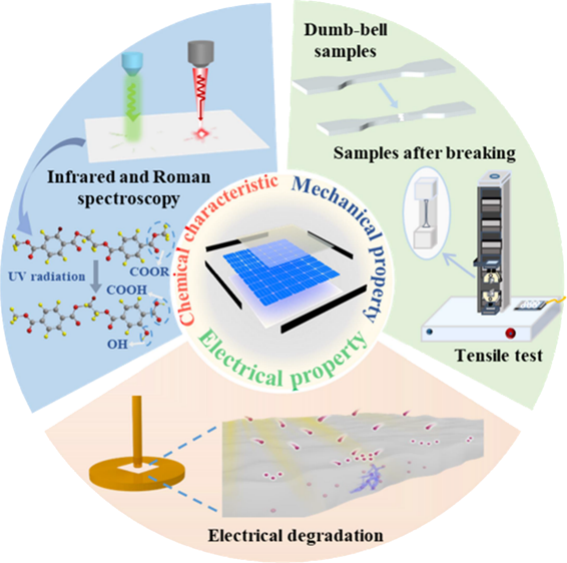

Photovoltaic (PV) power generation plays a significant role with the increase of installed capacity of renewable energy. The effects of environmental stress on insulating backsheets have been considered as the main cause of failure in PV systems. However, traditional aging models are difficult to realize the comprehensive evaluation of the lifetime of insulating backsheets. In this paper, the analytical method of complex chemical degradation related to the insulation was replaced by a physics-based method to quantify the elongation at the break as a function of time corresponding to temperature and radiation. In contrast to traditional aging models, this model simply used one parameter, namely drop-off rate (*v*), to reflect the degradation of polymers under various environmental stresses. The effect of ultraviolet (UV) radiation on the model was considered. Moreover, the electrical degradation, chemical changes, and mechanical properties caused by UV radiation were investigated to provide the reference for the lifetime of evaluation. The research is significant for comprehensively evaluating the lifetime of insulating materials for PV systems and other power equipment.

## 1 Introduction

The recent global energy crisis has highlighted the urgent need for renewable energy, with particular attention to the use of solar energy, which is a major factor in the future zero carbon energy system.^1,2^ The use of photovoltaic (PV) power generation technology to develop zero carbon energy and prevent energy crisis has also attracted intensive research interest.^3,4^

**Figure 1:**
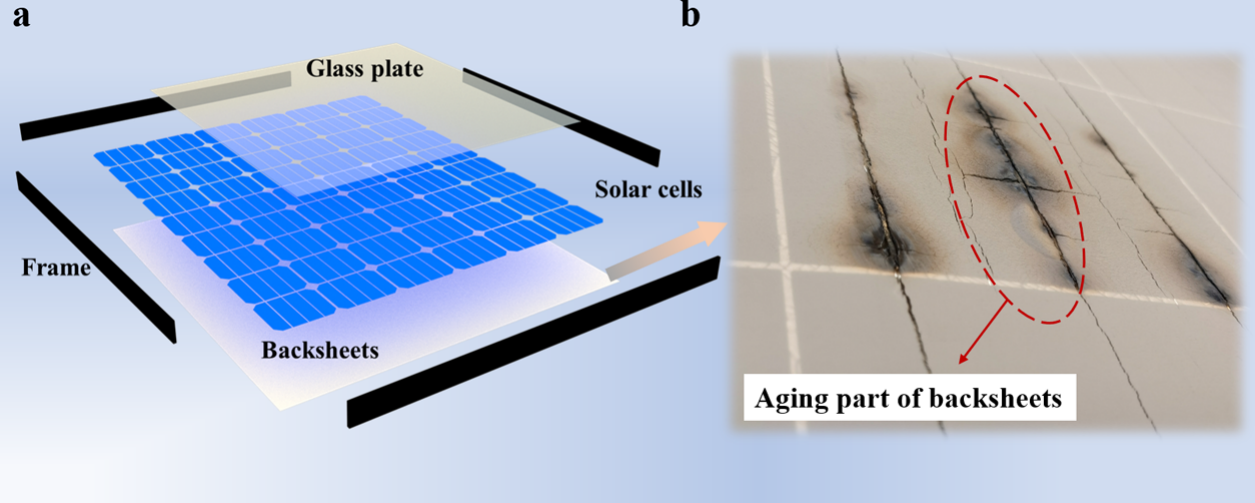
(a) Structure of the PV module. (b) Aging part of backsheets.

Figure 1a shows the structure of the PV modules. Backsheets are commonly used in PV modules for providing excellent electrical insulation and mechanical properties.^5−8^ PV backsheets always are subjected to UV light, heat, humidity, and so on, which can result in aging and performance loss,^9^ as shown in Figure 1b. These factors affect the performance of PV backsheets, casing that the expected lifetime of 25 years is unable to be guaranteed. Several research studies on the enhanced mechanical and UV protection polymers have been investigated to improve the lifetime of polymers.^10,11^ For instance, UV absorbers such as TiO_2_ and ZnO nanoparticles are used in the backsheets to provide the weather resistance. Synthesis of graphene sheets based on an economic and green method is developed to block harmful UV rays. It is vital to develop effective methods to evaluate the lifetime of polymers and provide reference for using renewable UV protective materials to improve the lifetime of polymers.

Presently, various kinetic models of chemical reactions have been proposed to characterize the polymer degradation.^12−14^ The chemical degradation related to insulation will occur in the polymer during aging, including chain scission, cross-linking, and oxidation. However, analyzing and investigating the effect of each reaction to the degradation is a complex problem. Thus, developing a mechanical model based on elongation at the break (EAB) property combining the reference data from the chemical reaction is a critical path to evaluate the lifetime of the polymer systematically.

Several research studies on degradation mechanisms of polymers subjected to various conditions, such as temperature, humidity, salt-mist, and electrical stress, have been investigated.^15−17^ As a predominant environmental factor, UV radiation highly affects the performance of insulating materials. It can embrittle the polymer and cause the loss of the mechanical property of backsheets, affecting the stability and the lifetime of the entire PV modules.^18^ Thus, the effect of UV radiation on the lifetime of backsheets should be considered.

In this paper, a mechanical model based on EAB considering the drop-off rate was proposed to quantify the degradation of polymers, and the influence of UV radiation on the aging model was verified. Moreover, the electrical degradation and chemical changes caused by UV radiation had been investigated to provide the reference for the lifetime of evaluation.

## 2 Materials and Experiments

**Figure 2:**
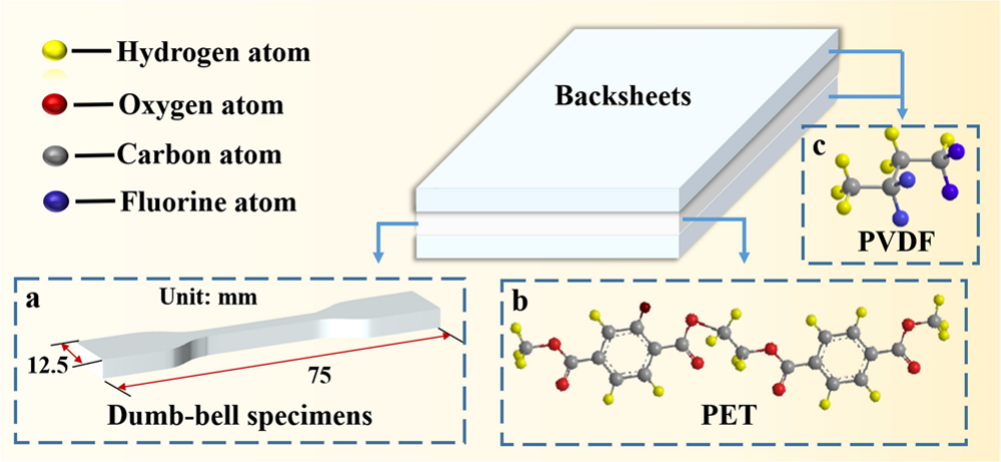
(a) Dumb-bell specimens of polyethylene terephthalate (PET) films. (b) Structure of repeat unit of PET. (c) Structure of repeat unit of polyvinylidene fluoride (PVDF).

PET and PVDF/PET/polyvinylidene fluoride (KPK) backsheets were selected to investigate the degradation behavior after UV exposure. Figure 2b,c shows the structures of repeat unit of PET and KPK. KPK backsheets consist of three laminates. The inner and outer layers are PVDF, and the core layer is PET. Prior to the experiments, all the tested PET films and KPK films were washed using absolute alcohol and then dried by an ionizing air blower. The degradation characteristics of the backsheets under UV radiation with different conditions were studied. The two accelerated conditions are (1) cyclic exposure of UVA-340 lamps at 0.76 W/m^2^/nm at 60 °C for 8 h and condensing humidity at 50 °C in the dark for 4 h and (2) continuous UVA-340 lamps at 0.80 W/m^2^/nm with a black panel temperature of 55 °C for 1000 h according to IEC TS 62788-7-2:2017. The PET and KPK films subjected to cyclic exposure of UVA-340 lamps for 0 cycle and 8 cycles were labeled as PET-1 and PET-2, KPK-1, and KPK-2, respectively. The PET and KPK films subjected to continuous UVA-340 lamps for 1000 h were marked as PET-3 and KPK-3. The dimensions of PET and KPK films in this paper were 150 × 75 × 0.19 and 150 × 75 × 0.32 mm. The chemical changes of films after UV exposure were characterized by infrared spectroscopy and Raman spectroscopy. The changes of crystallinity of backsheets after UV exposure were studied by differential scanning calorimetry (DSC). The mechanical performance was tested by a tensile test. To evaluate electrical properties, untreated and treated samples were subjected to partial discharge (PD) for 1 h. The applied voltages for PET and KPK were 1050 and 1140 V, respectively, which were set at 10% above the PD inception voltage value.

## 3 Results and Discussion

### 3.1 Mechanical Properties

The mechanical performance of polymeric materials is commonly determined by the EAB and tensile strength (TS).^19^ Changes in the mechanical property of the PET samples were tested by a tensile test. The extension rate of test was 10 mm/min. As shown in Figure 2a, the PET samples were made into dumb-bell specimens by a manual punching machine. The breakpoints within the gauge length marked on samples are considered valid. The experimental data reported here are averages of five samples.

**Figure 3:**
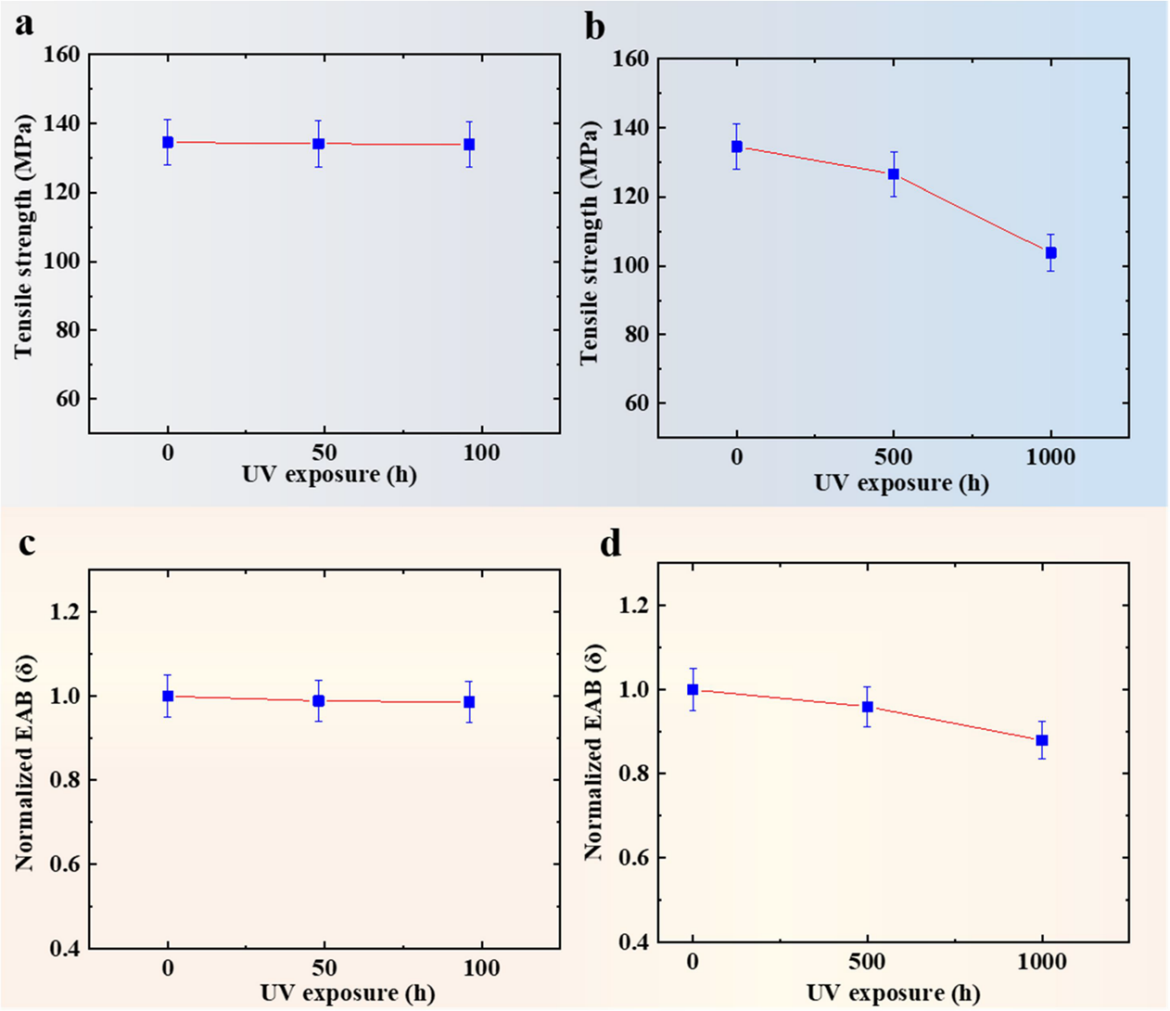
(a) TS for PET subjected to UV exposure after eight cycles. (b) TS for PET subjected to UV exposure for 1000 h. (c) Normalized EAB for PET subjected to UV exposure after eight cycles. (d) Normalized EAB for PET subjected to UV exposure for 1000 h.

**Figure 4:**
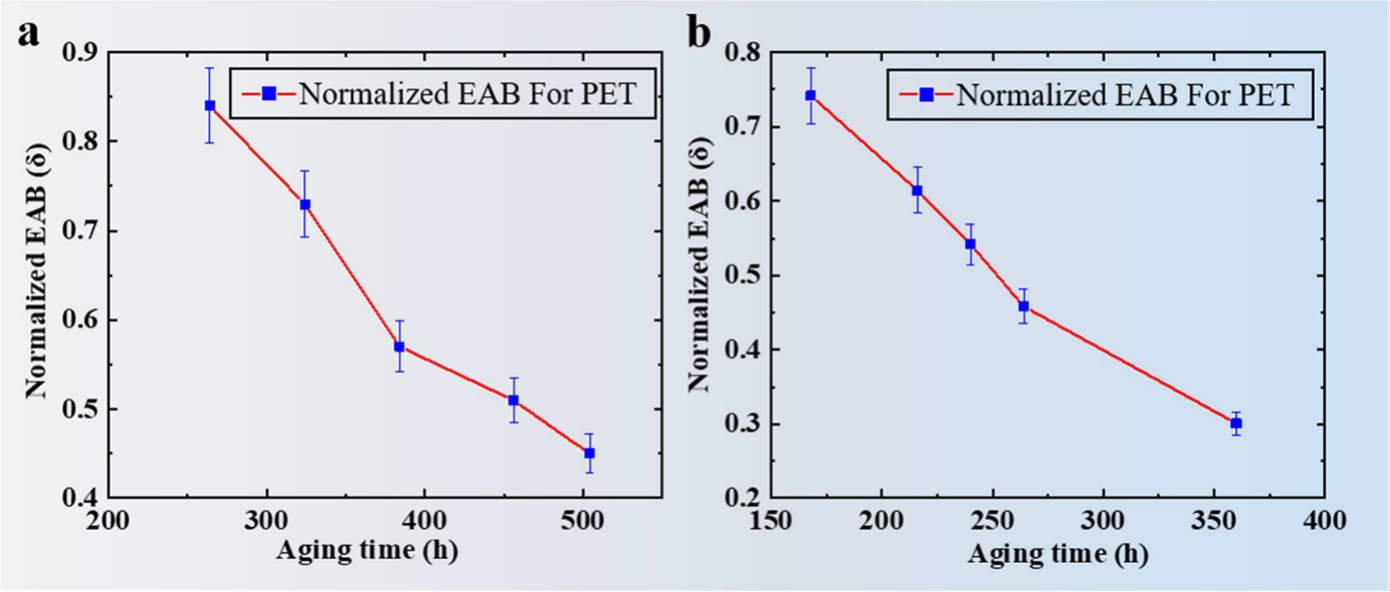
(a) Normalized EAB for PET subjected to temperature. (b) Normalized EAB for PET subjected to temperature and UV radiation.

Figure 3 shows the changes of TS and normalized EAB (EAB%) for PET after UV exposure. The EAB of PET before aging is 107.6%. The mechanical performance of PET after UV exposure for eight cycles changes slightly. After UV radiation for 1000 h, the TS of PET decreases from 134.5 to 103.8 MPa, and the EAB% decreases from 100 to 88%. The decrease of EAB% can be related to the chain scission. The decrease in performance is directly caused by the reduction of molecular mass. When the polymer film is tested in the machine direction, the polymer chain is completely oriented in the load direction after overtaking the yield stress. The load is absorbed by the valence bonds of the molecular chain instead of the intermolecular force. Consequently, the EAB is determined by the length and entanglement of the polymer chain. Higher molar mass of polymers can form longer chains and more tangles.^20,21^ During the photolysis of PET, the decrease of molar mass, the released volatile products, and the generation of carboxyl end-groups are discovered due to the chain scission.^5^ Thus, chain scission caused by UV degradation leads to the decrease in the EAB. The lifetime of polymers can be reached when the mechanical performance drops to 50% of the initial properties.^22^ To study the influence of UV radiation on the service life of backsheets, untreated samples and samples after UV exposure were treated at 150 °C for accelerating aging. Figure 4 shows the normalized EAB for PET subjected to temperature and UV radiation. Compared with the untreated samples, the EAB% of backsheets after UV radiation quickly decreases to 50% at the same aging temperature.

In addition, a mechanical model based on EAB considering the drop-off rate was proposed to quantify the degradation of polymers, and the effect of UV radiation on the model was considered. The normalized EAB (EAB%) after aging can be expressed by eq 1.^17,23^ The normalized EAB is represented by the δ in eq 1. The drop-off rate (*v*) is determined by the degradation of EAB rather than the measurement of the chemical reaction.
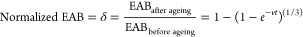
1

When the degradation process of the polymer at the initial stage decreases insignificantly, eq 1 should be transformed into eqs 2 and 3. Eq 2 reflects the *v* of the EAB begins after the end of *t*_0_. Eq 3 means that the drop-off of the EAB is considered negligible during the initial stage of aging.
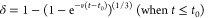
2
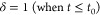
3where δ is normalized EAB, *v* is the drop-off rate whose unit is [1/time], *t* is aging time, and *t*_0_ is incubation time.

To simplify the fitting curve, eq 2 can be transformed into eq 4.
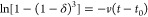
4*y* is set as the dependent variable to fit the experimental data.
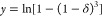
5

Eq 5 can be further transformed into eq 6 to determine the values of *v* and *t*_0_ from fitting results.
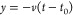
6

**Figure 5:**
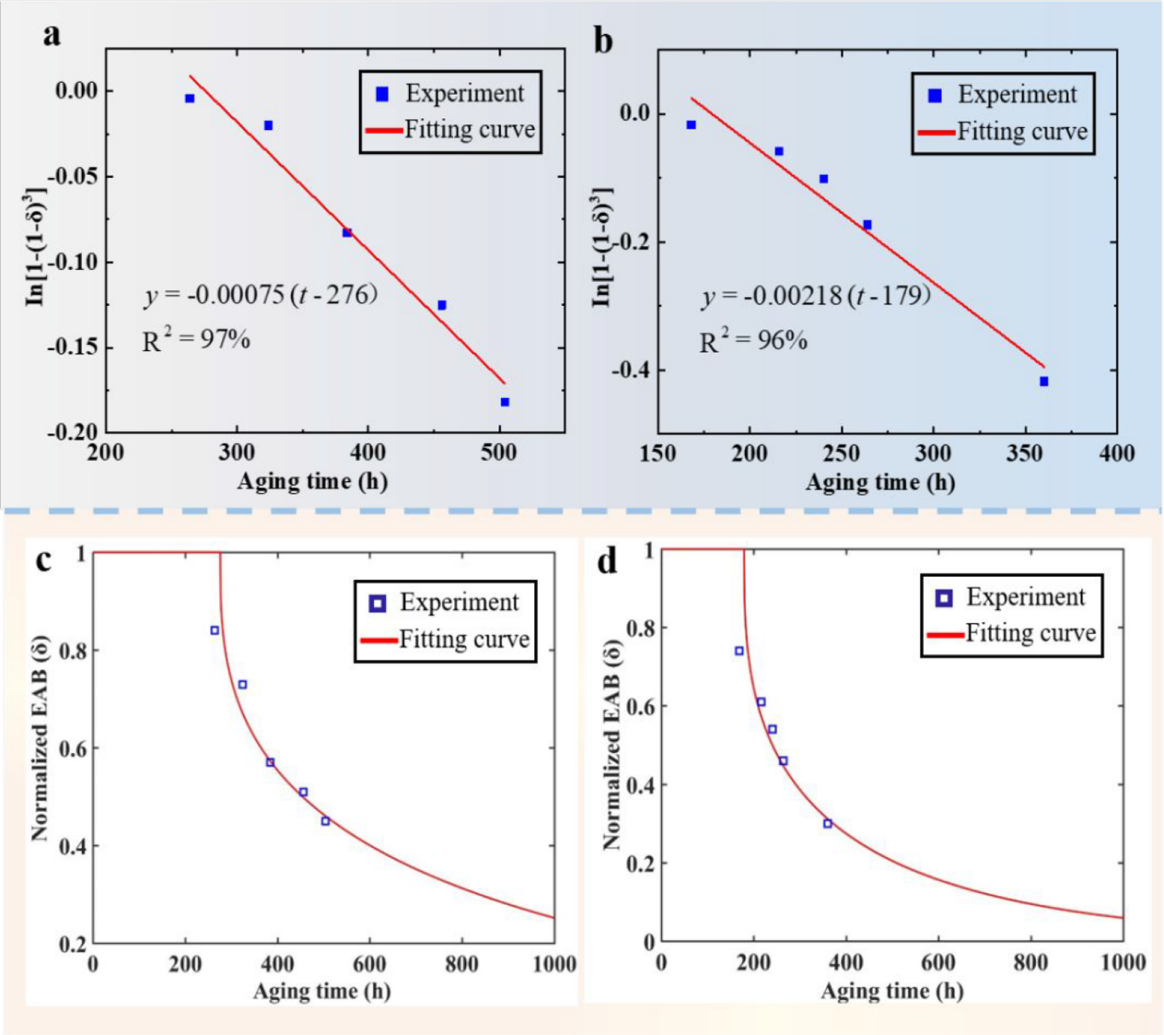
(a) Fitting curve for determining the parameters of the mechanical model affected by temperature. (b) Fitting curve for determining the parameters of the mechanical model affected by temperature and radiation. (c) Normalized EAB based on the mechanical model affected by temperature. (d) Normalized EAB based on the mechanical model affected by temperature and radiation.

As shown in Figure 5a,b, the experimental datas were used to fit curve to identify the values of *v* and *t*_0_ in eq 6. Table 1 shows the values of *v* and *t*_0_. By substituting different values of *v* and *t*_0_ into eqs 2 and 3, the continuous lines were drawn to fit the experimental data, as shown in Figure 5c,d. The results show that *t*_0_ becomes shorter and *v* becomes larger with the increase of UV radiation at the same aging temperature. At the initial stage of aging, the decrease of EAB is insignificant. Thus, the reduction of EAB in this period of time can be considered negligible. The *v* of EAB% under UV radiation and thermal treatment increases from 7.5 × 10^–4^ to 21.8 × 10^–4^, and the *t*_0_ decreases from 276 to 179 h, compared with the thermal treatment. UV radiation accelerates the aging process of the polymer at the initial stage. Therefore, UV radiation is a major factor to affect the lifetime of backsheets, which should be considered when estimating the service life of the PV module.

**Table 1:** Parameters of eq 6 for Figure 5

sample	UV exposure (h)	temperature (°C)	*v* (1/h)	*t*_0_ (h)
PET	0	150	7.5E–4	276
	1000	150	21.8E–4	179

### 3.2 Chemical Modification Analysis by Infrared Spectroscopy and Raman Spectroscopy

**Figure 6:**
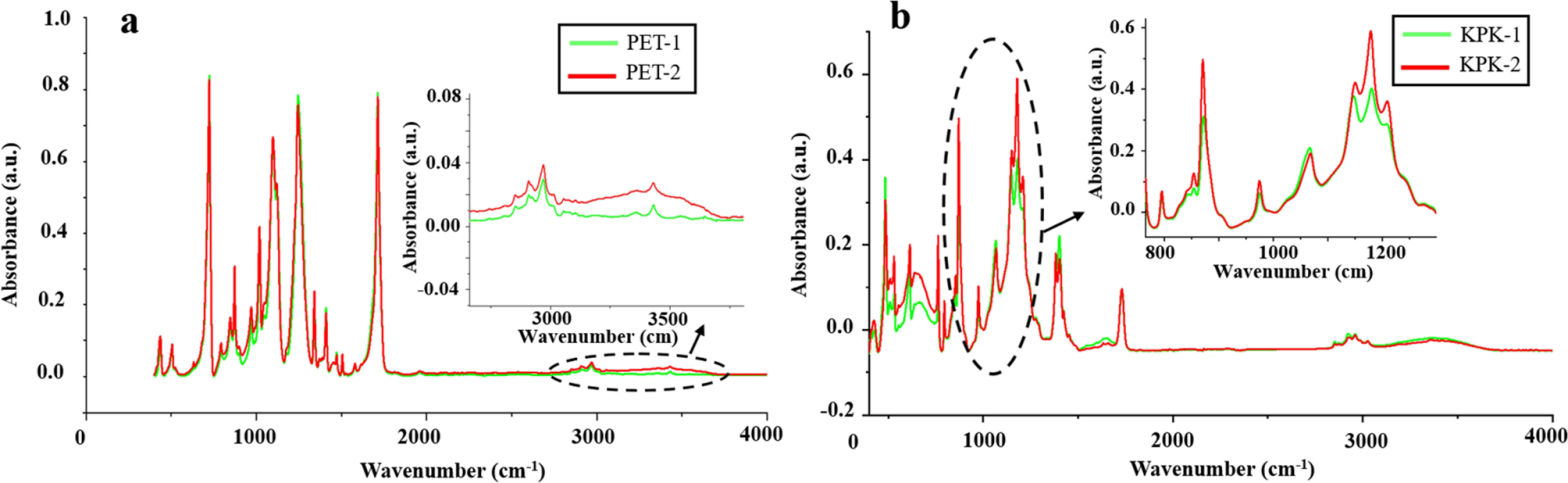
Infrared (IR) spectra of (a) PET films and (b) KPK films.

The chemical degradation of backsheets after UV radiation was studied by IR spectroscopy. Figure 6a shows the part of the IR spectra of PET samples treated by UV exposure. The peak at 2928 cm^–1^ is attributed to the C–H stretching vibration related to the Ar–CH_3_. An Ar–CH_3_ group can be generated by combining the phenyl and methyl radicals from the photolysis of PET during UV exposure.^5^ Peaks at 1578 and 1506 cm^–1^ are related to amorphous and crystalline ring stretching.^24^ The changes of peaks at 3550 and 3620 cm^–1^ are owing to the O–H stretching vibration. Figure 6b shows the part of the IR spectra of KPK films. PVDF is used in the outer layer of KPK films, and each repeating unit of PVDF carries two fluorine atoms. The characteristic peak at 762 cm^–1^ is noticed, which is attributed to CF_2_ bending. The peak at 1178 cm^–1^ is related to CF_2_ symmetric stretching.^25^ The peak at 1731 cm^–1^ can be related to the amorphous and crystalline carbonyl stretching.

**Figure 7:**
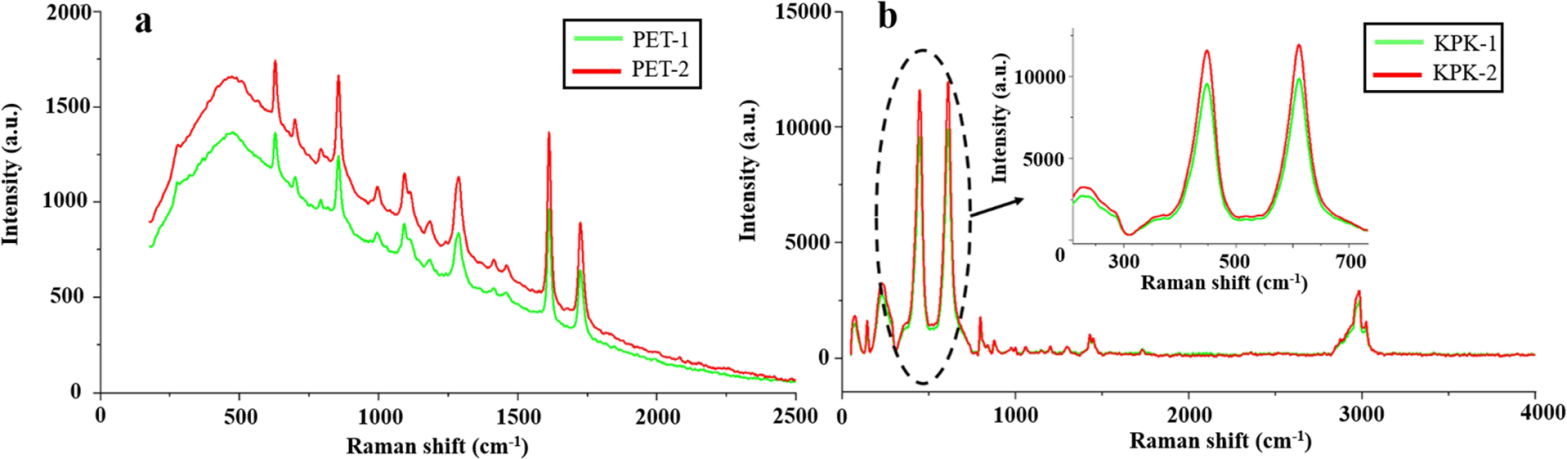
Raman spectra of (a) PET films and (b) KPK films.

The Raman images of the PET and KPK films after UV exposure are displayed in Figure 7. Compared with the untreated samples, the intensity of the Raman peak for irradiated samples increases, which indicates that some chemical changes occur in PET’s surface due to UV exposure. The peaks at 861 and 1728 cm^–1^ are related to COO bending vibration and C=O stretching vibration. The variation of the Raman shift at 1614 cm^–1^ is interrelated to ring C=C stretching, which is related to the strong aromatic character of PET. Carboxylic acid and other molecules can be generated by the ether bond cleavage of the ester groups for PET during the photodegradation process.^26^Figure 7b shows the Raman spectra of KPK films. PVDF is used in the outer layer of KPK films, and each repeating unit of PVDF carries two fluorine atoms. Figure 7b shows the Raman peaks of fluorine-related bands. The Raman peaks at 450 and 614 cm^–1^ are related to TiO_2_, which is a common inorganic additive used in fluoropolymer backsheets. The Raman peak at 1284 cm^–1^ can be related to CF_2_ asymmetric stretching. CF stretching vibration is observed at 1372 cm^–1^ and CF_2_ asymmetric stretching is recorded at 1300 cm^–1^.^25^

**Figure 8:**
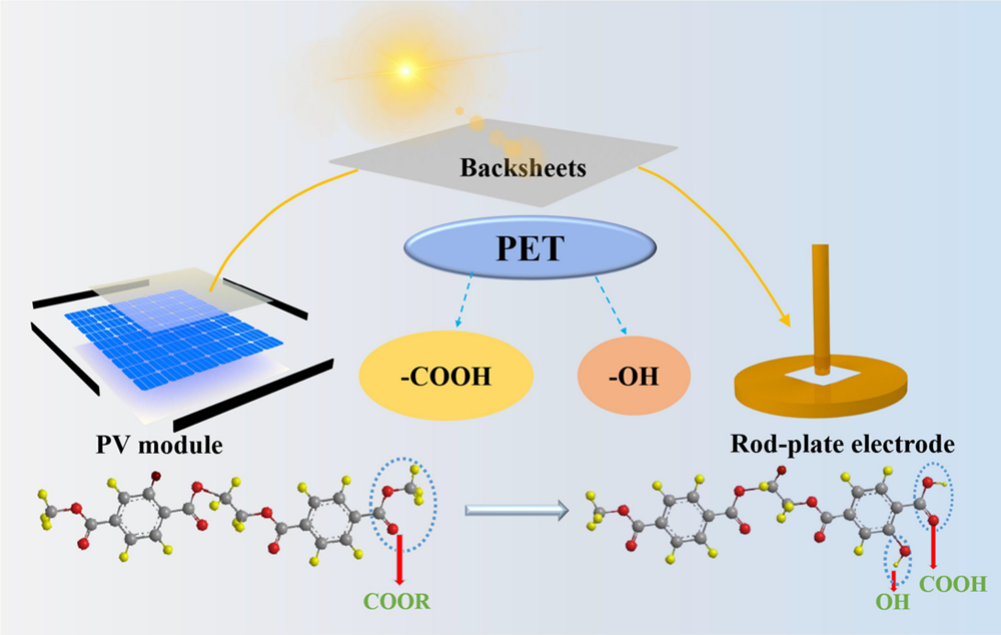
Chemical products of PET films after UV irradiation.

Figure 8 shows the chemical products of PET after UV irradiation. The photodegradation process of PET includes purely photolytic chemistry and photo-oxidative reactions. PET can be degraded during the photodegradation, which leads to the reduction in molar mass, generating volatile products and −COOH.^27^ From a molecular perspective, the olefin and the corresponding acid can be produced by an intramolecular rearrangement of aliphatic and aromatic esters with a γ-hydrogen atom. In addition, PET will undergo an oxidative reaction sequence in air during the UV exposure. ROO· can be produced by the oxidative reaction of alkyl radicals induced by photolysis process. Subsequently, the above react further produces volatile gas during photo-oxidative reactions.^5^

### 3.3 Thermoanalytical Investigations

The DSC measurements were used to determine the temperature characteristics. The measurements were made in a temperature range of 30–350 °C with a heating rate of 10 °C/min. The crystallization behaviors of PET films were characterized by DSC to explore the effects of UV radiation on the crystallization of polymers.

The crystallinity *X*_c_ is expressed by eq 7:
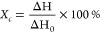
7where Δ*H* is the melting enthalpy, and Δ*H*_0_ is the melting enthalpy of the polymer fully crystallized.

**Figure 9:**
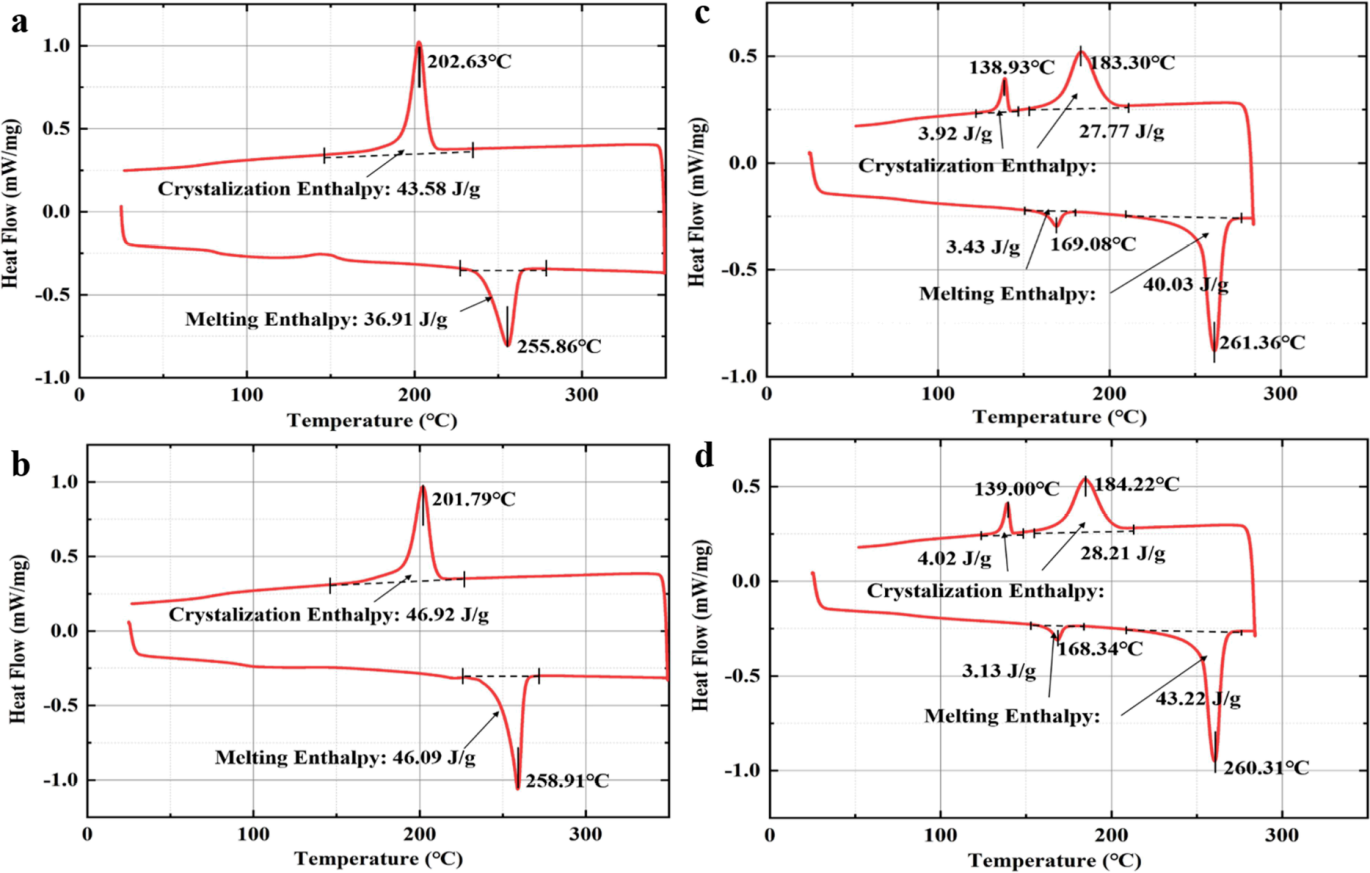
DSC curves of (a) PET-1, (b) PET-2, (c) KPK-1, and (d) KPK-2.

Figure 9 shows the DSC curves of the untreated and treated films. The melting peak of PET films is between 225 and 275 °C with a peak temperature of 255.86 °C. The melting enthalpy can reflect the change of crystallinity during aging. Table 2 shows the melting and crystallization parameters of samples. The results clarify that the melting enthalpies of samples marked as PET-1 and PET-2 are 36.91 and 46.09 J/g, which indicates that the Δ*H* of PET increases after UV exposure. The Δ*H* of fully crystallized PET is 140 J/g.^28^ The crystallinity of PET after UV exposure increases from 26.4 to 32.9%.

**Table 2:** Melting and Crystallization Parameters of Samples

sample	Δ*H* (J/g)	*T*_m_ (°C)	*X*_c_ (%)
PET-1	36.91	255.86	26.4
PET-2	46.09	258.91	32.9
KPK-1	40.03	261.36	28.7
KPK-2	43.22	260.31	30.9

Figure 9c,d shows that the DSC curves of the unaged KPK backsheets reveal two melting peaks, which is attributed to the PVDF-PET-PVDF structure of KPK films. The melting temperature (*T*_m_) of PVDF is between 150 and 175 °C with a peak temperature of 169.08 °C. The melting temperature of PET is between 200 and 300 °C with a peak temperature of 261.36 °C. The change of melting enthalpy of PET in KPK films was calculated to explore change of crystallinity. The melting enthalpy of PET in the PVDF-PET-PVDF structure increases from 40.03 to 43.22 J/g, and the crystallinity increases from 28.7 to 30.9%. The melting enthalpy of backsheets increases during aging, which indicates the presence of the crystallization process induced by UV radiation. This process can be related to the chain scission in the amorphous phase. The entangled chain segments released by chain scission can become sufficiently free to find a spatial rearrangement into the crystalline phase.^15,29^ Compared with the traditional single-layer PET backsheets, the crystallinity of KPK changes relatively lower. This can be attributed to excellent weather resistance and protection provided by the PVDF layer of the multilayer KPK.

### 3.4 Electrical Degradation Induced by PD

**Figure 10:**
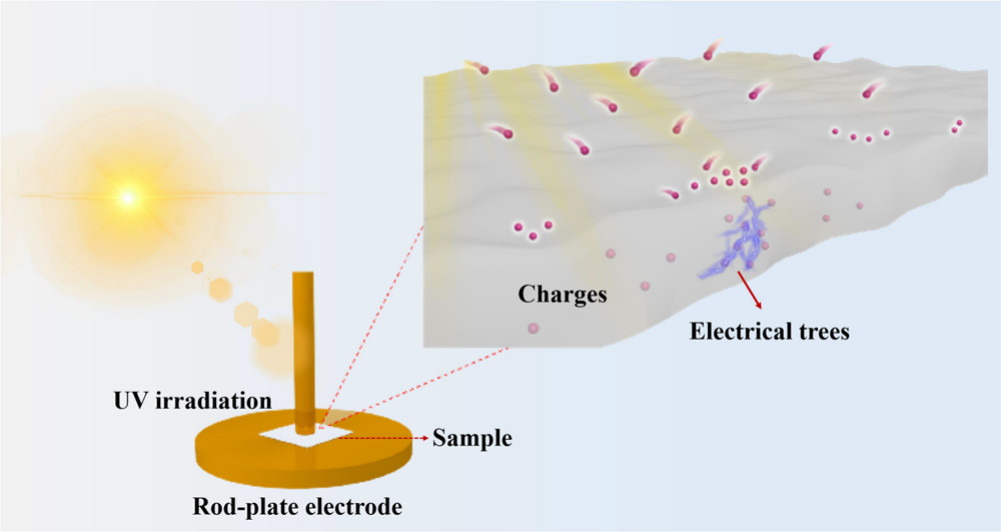
Electrical degradation of backsheets after UV radiation.

**Figure 11:**
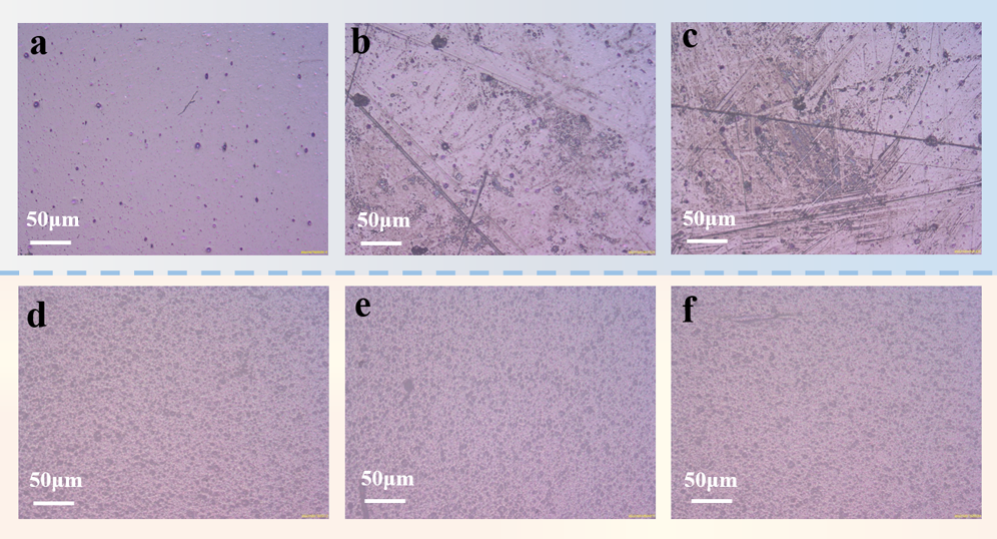
Surface topography of samples. (a) Untreated PET films. (b) PET films after UV radiation. (c) PET films after UV radiation and PD stress. (d) Untreated KPK films. (e) KPK films after UV radiation. (f) KPK films after UV radiation and PD stress.

**Figure 12:**
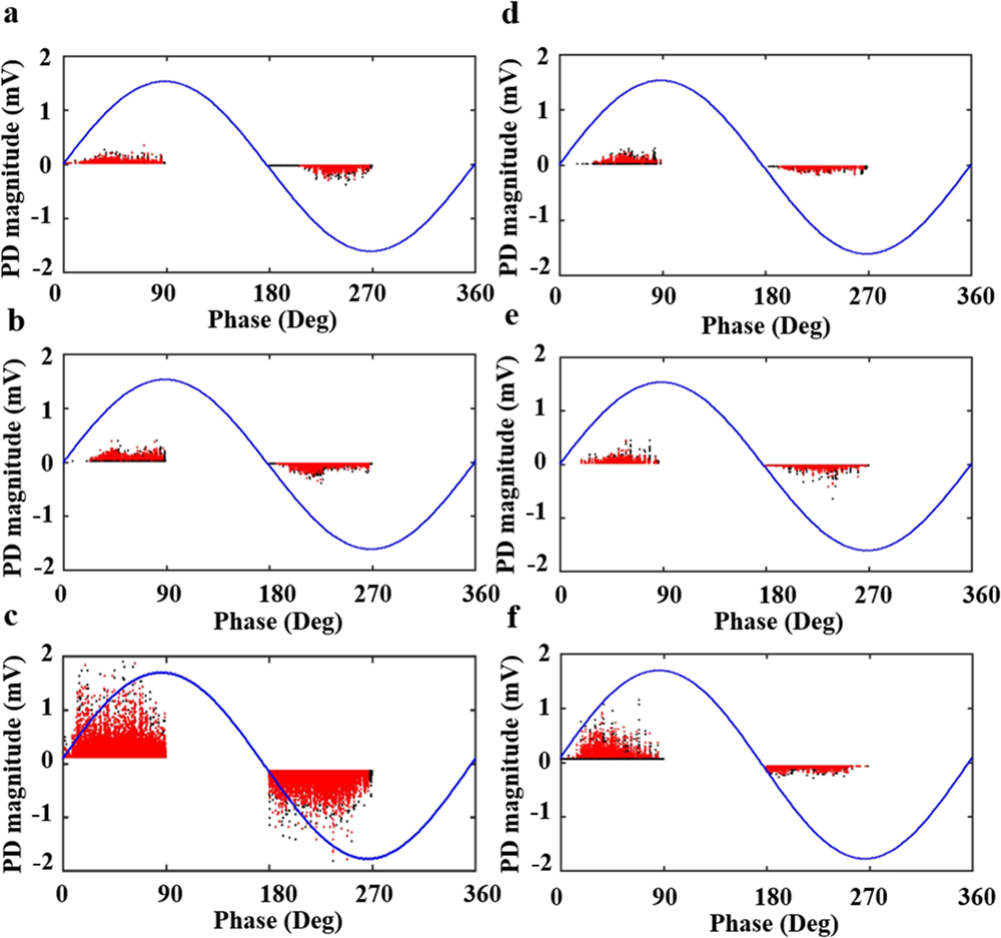
Patterns of PRPD of (a) PET-1, (b) PET-2, (c) PET-3, (d) KPK-1, (e) KPK-2, and (f) KPK-3.

To investigate the electrical properties after UV exposure, all samples were subjected to PD for 1 h. The PD experiments were conducted in the air, and the temperature and the relative humidity were in the range of 24.6–26.7 °C and 39–47%, respectively. Figure 10 shows the electrical degradation of backsheets after UV irradiation. Phase-resolved partial discharge (PRPD) patterns are used to explore the electrical degradation properties of PET and KPK backsheets after UV irradiation. The PD intensity is related to PD amplitude, as well as the PD repetition rate.^30^ The chain scission process during the photolysis of polymer can result in the decrease in molar mass, volatilization of degradation products and generation of polar radicals, leading to the erosion of the backsheets.^27^Figure 11 shows the surface topography of untreated samples and samples after aging. The results show that UV radiation erodes the surface topography of PET. Figure 11d,e shows that UV radiation has slight effect on the surface of KPK backsheets. Figure 11b shows the surface topography of PET samples after 1000 h of UV radiation. There are some fine cracks on the PET surface due to the erosion of the backsheets and the decrease in mechanical properties. Severe UV radiation can accelerate the degradation process of the polymer, causing more erosion and defects.^27^Figure 12 shows that the PD events have a phase angle between 0° and 90°, 180°, and 270°. The red region in the PRPD pattern represents more dense discharge part compared with the black region. PRPD patterns show that the PD events of UV-treated backsheets are more dense than untreated films. With the degradation of polymeric materials, the thickness of the polymer decreases continuously, which can result in the production of pits. The accumulation of charges in these pits can effectively improve the probability of initial electrons, which can easily generate PD and lead to stronger discharge.^31−33^Figure 11c shows the surface topography of PET after UV radiation and PD stress. Some burn marks exist on the surface of PET films, which may be related to the bombardment from high-energy ions and high temperature generated by discharge during PD. After PD for a long period, the growth of electrical trees may ultimately cause the dielectric breakdown of insulating polymer.^33^

Compared with the traditional single-layer backsheets, the discharge of KPK backsheets is relatively lower (Figure 12). The interface between the PVDF layer and PET layer may hinder the generation of discharge channels, which enhances the electric breakdown strength of KPK films.^34^ Furthermore, the PVDF of the outer layer is a fluoropolymer formed of C–H and C–F bonds. The C–F bond has high dissociation energy and high electronegativity. It makes PVDF have excellent thermal stability, chemical, oxidation resistance, and mechanical stability, especially resistance to the outdoor environment, which can slow the degradation process of the polymer induced by UV radiation.^35^

## 4 Conclusions

The investigation on the aging of backsheets is significant for evaluating the service life of the PV system. The results based on the mechanical model indicate that the drop-off rate (*v)* of EAB% after UV radiation and thermal treatment increases from 7.5 × 10^–4^ to 21.8 × 10^–4^ compared with the single thermal effect. UV radiation accelerates the degradation process of polymer at the initial stage, which increases the drop-off rate (*v*) of EAB% until it reaches the useful lifetime of the polymer. It can be concluded that UV radiation has considerable influence on evaluating the service life of PV modules. The electrical degradation, chemical changes, and mechanical properties caused by UV radiation have been investigated to provide the reference for the lifetime of evaluation. The research of degradation evaluation based on the mechanical model provides a significant reference for evaluating the lifetime of insulating materials for PV systems and other power equipment.
